# Inflammation in cancer and depression: a starring role for the kynurenine pathway

**DOI:** 10.1007/s00213-019-05200-8

**Published:** 2019-02-26

**Authors:** Luca Sforzini, Maria Antonietta Nettis, Valeria Mondelli, Carmine Maria Pariante

**Affiliations:** 1 Psychiatry Unit, Department of Biomedical and Clinical Sciences, ASST Fatebenefratelli-Sacco University Hospital, Università di Milano, Milan, Italy; 2 Institute of Psychiatry, Psychology and Neuroscience, Department of Psychological Medicine, King’s College London, London, UK; 3 National Institute for Health and Research Biomedical Research Centre at South London and Maudsley NHS Foundation Trust and King’s College London, London, UK

**Keywords:** Cancer, Chemotherapy, Depression, IDO inhibitors, Immunotherapy, Inflammation, Indoleamine 2-3-dioxygenase, Kynurenine

## Abstract

Depression is a common comorbidity in cancer cases, but this is not only due to the emotional distress of having a life-threatening disease. A common biological mechanism, involving a dysregulated immune system, seems to underpin this comorbidity. In particular, the activation of the kynurenine pathway of tryptophan degradation due to inflammation may play a key role in the development and persistence of both diseases. As a consequence, targeting enzymes involved in this pathway offers a unique opportunity to develop new strategies to treat cancer and depression at once. In this work, we provide a systematic review of the evidence up to date on the kynurenine pathway role in linking depression and cancer and on clinical implications of this evidence. In particular, complications due to chemotherapy are discussed, as well as the potential antidepressant efficacy of novel immunotherapies for cancer.

**Electronic supplementary material:**

The online version of this article (10.1007/s00213-019-05200-8) contains supplementary material, which is available to authorized users.

## Introduction

Depression and cancer are both leading causes of death and disability worldwide, and they often co-occur together, with a consequent heavy socioeconomic burden. In 2017, it was estimated that more than 300 million people were suffering from depression, with rising numbers, as shown in more than 18% increase in a 10-year range, between 2005 and 2015. Depression is associated with an increased mortality risk compared to general population (Mols et al. 2013; Cuijpers et al. 2014; Gilman et al. 2017). Indeed, it can lead to death by suicide, as its worst manifestation. It is widely known that cancer, with all its subtypes, is one of the biggest challenges for modern medicine and public health, accounting for around 15–20% of the global annual deaths, with this rate significantly increasing year on year (Wang et al. 2016a). Depression prevalence rates in cancer patients range from 1% to above 50% (Massie 2004), with differences due to different diagnostic measures used to assess depression (Krebber et al. 2014) and to cancer type, stage, treatment, and disease course. Moreover, depression in cancer is difficult to assess, especially due to the high overlap of neurovegetative symptoms between these two diseases: for example, fatigue, sleep alterations, and reduced appetite. Despite this variability, it is now clear that patients with cancer are a population at greater risk for the development of clinically relevant depressive syndromes, with overall higher prevalence rates than those observed in general population (Miller et al. 2008; Mitchell et al. 2011; Bortolato et al. 2017). Indeed, depression rates are highest (up to 30–50%) in specific types of cancer, like pancreatic, oropharyngeal, and breast cancer (McDaniel et al. 1995) and contribute to worsen the disease course (Sotelo et al. 2014). On the other hand, evidence suggests that a mood disorder can precipitate the course of chronic inflammatory diseases, including cancer (Balon 2006), implying that there is a bidirectional link connecting cancer and depression. Despite this evidence, the underlying biological mechanism linking these two conditions is still unknown.

In the last decades, increasing evidence has been gathered on the presence of an immune dysregulation in both depression and cancer (Zalli et al. 2016). As a consequence, it has been hypothesized that immune activation and inflammation may play a key role in the pathophysiology of both diseases. Several studies have explored the inflammatory pathway to depression. Such pathway appears to partially involve the communication between peripheral and central immune system (CNS). In fact, peripheral inflammatory cytokines have been shown to affect the brain through different ways (Cattaneo et al. 2015). This finally results in the activation of the CNS immune cells, like microglia, the brain resident macrophages. In turn, activated microglia promote central inflammation, which affects neuroplasticity, neurogenesis, and neurotransmitter metabolism, leading to behavioral changes typical of depression (Brites and Fernandes 2015; Bhattacharya et al. 2016). Thus, cancer, as well as other chronic diseases, could lead to the development of depressive symptoms by inducing inflammatory responses and acting on the immune signaling to the brain (Pyter et al. 2009; Walker et al. 2013; Lebeña et al. 2014; Yang et al. 2014). In particular, the kynurenine pathway has been suggested to play a key role in this process and to be the link between the two diseases.

Despite the relatively recent raising interest, the kynurenine pathway was first described in the middle of the nineteenth century, more than 165 years ago. It is the catabolic process in which tryptophan is converted to kynurenine instead of serotonin. Tryptophan is an essential amino acid, crucial in brain homeostasis, as it is the precursor to the neurotransmitter serotonin and the hormone melatonin. However, the major route for tryptophan catabolism in mammalian cells is the kynurenine pathway. In this pathway, tryptophan is converted to kynurenine and is degraded leading to the production of nicotinamide adenine dinucleotide (NAD+) as the end product, which is necessary for energy production. Kynurenine and its further breakdown products are globally called “kynurenines.”

The first step of the process is catalyzed by two heme-dependent enzymes: tryptophan 2,3 dioxygenase (TDO) and indoleamine 2,3 dioxygenase (IDO) (1 and 2). TDO is predominantly expressed in the liver tissue, while IDO is diffused in most of the tissues in the human body. This last enzyme seems to be an important link between the kynurenine pathway and inflammation, because of its activation by pro-inflammatory cytokines. Proceeding through the metabolic cascade, another dioxygenase (3-hydroxyanthrenilic acid dioxygenase (HAO)) creates a compound (alpha-amino-beta-carboxymuconate-epsilon-semialdehyde) which decays non-enzymatically to quinolonic acid (QUIN), a NAD+ precursor. Another critical enzyme for QUIN production is kynurenine 3-monooxygenase (KMO).

It has been demonstrated that QUIN can selectively activate glutamate N-methyl-D-aspartate (NMDA) receptors and its accumulation could result in excitotoxicity to the neurons and disturb the glutamatergic transmission (Bender and McCreanor 1985). In an alternative pathway to the NAD+ production, QUIN can be converted into kynurenic acid (KYNA), which is demonstrated to be neuroprotective, counteracting the effects of QUIN. So, there is a balance between neurodegenerative and neuroprotective effects in the kynurenine pathway, expressed by the QUIN/KYNA ratio, which is strictly related to immune activation. This process involves the microglia and the astrocytes cells, which are the primary site for tryptophan catabolism in CNS. KYNA is mainly produced by astrocytes, while microglia are responsible for QUIN production. QUIN/KYNA, as well as the most used kynurenine to tryptophan ratio (K/T ratio), could be used as a measure of the pathway activity.

As mentioned above, the kynurenine pathway is strictly related to the immune response. Evidence suggests that inflammation significantly shifts tryptophan metabolism to kynurenine production, carrying out several biological functions. An increased kynurenine pathway activity may therefore be associated with various clinical conditions linked to immune activation, such as depression and cancer. Hence, it is conceivable that the kynurenine pathway could represent one of the links between depression and other clinical conditions such as cancer, even though the mechanisms are not always clear and still not completely understood. Thus, an overview of the current evidence on this topic and its clinical implications is needed.

The aim of this paper is to investigate the scientific evidence to date on the role of the kynurenine pathway in linking depression and cancer. While the background information on the kynurenine pathway and on the comorbidity between depression and cancer has been provided as a narrative review, we adopted a systematic approach to identify all the clinical and preclinical studies analyzing kynurenine pathway as a biological link between depression and cancer. We also consider the clinical implication of such association in terms of treatment strategies and we discuss the effects of current medications and of potential new therapeutic agents.

## Kynurenine pathway in depression

The limits of monoaminergic psychopharmacological therapies are well known to clinical practitioners. Treatment-resistant depression (TRD) is a high prevalence event, with rates of 12%–20% (Mrazek et al. 2014). This suggests that other factors than monoamine depletion could contribute to the pathogenesis of depression. As a consequence, in the last decades, alternative etiologies of major depressive disorder (MDD) have been investigated in order to identify new potential treatment targets (Réus et al. 2015), such as the immune system.

Compelling evidence suggests that inflammatory patterns, including abnormalities of the kynurenine pathway, may be implicated in the pathophysiology of depression (Savitz 2017). Animal models have shown that depressive-like behavior is associated with an increased inflammation and increased IDO activity (O’Connor et al. 2009; Norden et al. 2015). A similar association has been found in humans, where the kynurenine pathway activation has been associated with the development of depressive symptoms, and with symptoms severity. For example, QUIN/KYNA ratio is higher in depressed patients than in healthy controls (Myint 2012), suggesting an imbalance of kynurenine metabolites towards neurodegenerative effects. A study from Savitz et al. (2015b) indicates that these abnormalities persist also in the remission phase of MDD patients, diagnosed according to Diagnostic and Statistical Manual of Mental Disorders (DSM-IV-TR). A recent meta-analysis (Ogyu et al. 2018) revealed that depressed patients have decreased KYNA levels compared with healthy controls, while no difference was found in the levels of QUIN. Interestingly, they found increased QUIN levels, together with decreased KYNA levels, in antidepressant-free patients compared with healthy controls. Therefore, monoaminergic antidepressants, as well as other treatments such as omega-3 fatty acids, could exert their antidepressant activity by reducing kynurenine pathway–related neurotoxicity (Borsini et al. 2017). In terms of symptom domains, Hestad and colleagues (Hestad et al. 2017) showed K/T ratio to be particularly useful, given the specific association between elevated K/T ratio and poorer cognitive performance in depression.

A long-lasting and/or uncontrolled immune activation, as observed in multiple chronic diseases, has been associated with clinically relevant behavioral symptoms, such as depression (Capuron and Miller 2011), and the kynurenine pathway appears to be strictly implicated in this association. Indeed, there is vast evidence linking clinical conditions characterized by a chronic immune response (such as hepatitis C, autoimmune disorders, cardiovascular diseases, diabetes, and cancer) to the activation of the kynurenine pathway (Oxenkrug 2013; Walker et al. 2013; Sperner-Unterweger et al. 2014). Consequently, the kynurenine pathway could hypothetically mediate the development of depressive symptoms in the above-mentioned conditions. Hepatitis C is largely related to depressive symptoms, with very high depression prevalence rates. However, these data are influenced by cytokine immunotherapy, such as interferon (IFN)-alpha, that could in turn frequently cause depression (Schaefer et al. 2012), as discussed in the later section on “Implication for intervention.” Alterations of the kynurenine pathway could also mediate sleep disturbance, which is associated with elevated QUIN/KYNA ratio and increased C-reactive protein (CRP), as showed by Cho and colleagues in their interesting clinical study on depressed patients (Cho et al. 2017). In addition, several genetic alterations of the enzymes involved in this pathway have been identified and have been proposed to be associated with MDD, especially polymorphisms of the genes encoding IDO1/2 and KMO (Boros et al. 2018).

Finally, alterations of the kynurenine pathway were studied also with neuroimaging techniques. Magnetic resonance imaging (MRI) studies in depressed patients (identified according to DSM-IV-TR criteria) showed reductions in cortical thickness in depressed patients compared with healthy controls, particularly in right medial prefrontal cortex (mPFC), together with increased QUIN/KYNA ratios (Meier et al. 2016). Moreover, in MDD patients, kynurenine and K/T ratio were inversely associated with the total striatal volume, defined as the sum of the volumes of the nucleus accumbens, caudate, and putamen (Savitz et al. 2015a).

Nonetheless, there are discrepancies that should be taken into account. Indeed, some studies showed no correlation or even a negative correlation between kynurenine pathway activity and depression. Dahl and colleagues (Dahl et al. 2015) found no increase in kynurenine pathway plasma markers in patients with a depressive episode, assessed with DSM-IV criteria and the Montgomery–Åsberg Depression Rating Scale (MADRS, total score ≥ 20), compared with healthy controls. The kynurenine pathway was unaltered after a 12-week antidepressant treatment, which significantly reduced symptoms scores. Similarly, Baranyi and colleagues (Baranyi et al. 2017) did not find any difference in kynurenine and QUIN concentrations in depressed subjects compared with healthy controls. Still, they found lower KYNA concentrations in depressed patients, partially confirming the results discussed above. In a recent study, kynurenine levels and K/T ratio were actually decreased in MDD medication–free patients, again diagnosed according to DSM-IV criteria; interestingly, kynurenine levels were increased after 8 weeks of antidepressant treatment (Umehara et al. 2017). However, no information on the other metabolites was collected. Some data also suggest that the inflammatory phenotype present during depression might be independent from kynurenine pathway activation and IDO expression (Hughes et al. 2012). Similarly, depressive symptoms during inflammation might develop independently from tryptophan degradation (Quak et al. 2014). As Arnone and colleagues suggest in their interesting systematic review and meta-analysis (Arnone et al. 2018), such conflicting results may be due to various clinical variables and confounders, like age, sex, and metabolic status of participants, as well as illness severity, antidepressant treatment, study design, and power calculation. The lack of direct measurements of central inflammation, including kynurenine pathway markers, complicates even more our understanding of this relationship. Of note, it should be considered that depression during chronic inflammatory conditions could be etiologically different from other depressive disorders (Arnone et al. 2018) and that kynurenine pathway alterations could be present only in the specific subgroup of depressed patients with immune comorbidities (Miller and Raison 2016).

In conclusion, the majority of studies to date suggest that an overactivation of the kynurenine pathway might be an important intersection point between genetic and environmental factors involved in the pathophysiology of depression (Oxenkrug 2010). Moreover, IDO activity may be a valid target for new antidepressant treatments. However, it seems clear that additional and more tailored research is needed to strengthen our knowledge.

## Kynurenine pathway in cancer

Given the importance of tryptophan metabolism and kynurenine pathway in driving inflammatory responses, it is not surprising that the kynurenine pathway has recently emerged as an important factor in the pathogenesis of many types of cancer. In addition, tryptophan metabolism is also critical for cell proliferation and immunoregulation. What is still unclear is whether the kynurenine pathway in cancer is activated by the biology of the disease (for example inflammatory processes) or simply by the related stress. However, its role in tumorigenesis needs particular attention and has been highlighted by several findings.

First, recent evidence suggests that IDO activity may support the tumor escape from the immune system (Gostner et al. 2015). There are two types of IDO enzyme, IDO1 and IDO2, that convert tryptophan to kynurenine, with different activity rates. IDO2 is less expressed and has a weaker enzymatic activity than IDO1. IDO1 activity is strictly related to two key inflammatory cytokines, interferon-gamma (IFN-gamma) and interleukin 6 (IL-6), whose function in inflammation and in cancer is widely known even if not completely understood: the first one has a prevalent anti-tumoral effect, the second one is mainly a pro-tumorigenic factor. IFN-gamma activates IDO1 expression, which in turn activates IL-6, creating a network where IDO1 acts like a negative feedback on IFN-gamma, and partially upregulates IL-6. Being a regulatory interface between IFN-gamma and IL-6, IDO1 promotes a pro-inflammatory response that plays a role in cancer neovascularization, by enhancing new blood vessel development (Prendergast et al. 2017). More importantly, this enzyme seems to promote tumor progression, by predominantly acting on regulatory T cells (Tregs), effector cytotoxic CD8+ T cells (Teff), and natural killer cells (NK) (Munn and Mellor 2016); these lymphocytes exert a protective activity by reducing tumor progression and improving antitumor immunity, but kynurenine can suppress CD8+ T and NK cells and bias Tregs differentiation (Muller et al. 2018). This activity is proved to be reversible by administration of a therapeutic enzyme, PEGylated kynureninase, that degrades kynurenine to an immunological inert compound, with a related marked increase in proliferation of CD8+ lymphocytes, that in turn promote tumor infiltration (Triplett et al. 2018). Although less studied, IDO2 has been found to be overexpressed in some human tumors, to functionally enable IDO1-dependent Treg suppression (Metz et al. 2014), and to underpin B cell–mediated autoantibody production (Merlo et al. 2016), which is important in the development of certain cancers, such as squamous cell carcinomas (Prendergast et al. 2017). Together, the *IDO1* and *IDO2* genes are variably upregulated in neoplastic cells as well as in stromal, endothelial, and innate immune cells of the tumor microenvironment and in tumor-draining lymph nodes.

Data are available also on the other tryptophan-catabolizing enzyme, TDO, that is constitutively expressed in the liver and responsible for metabolizing dietary tryptophan. TDO is also activated during cancer. From recent findings, gene expression levels of TDO2, the gene encoding TDO, correlate with poorer breast cancer clinical outcomes (Greene et al. 2018). All together, these findings suggest that new pharmacologic agents may target both IDO (1 and 2) and TDO.

The dysregulation of the kynurenine pathway in cancer may also promote malignancy by NAD+ production, which could directly affect several cellular functions. Furthermore, NAD+ can activate the transcription factor aryl hydrocarbon receptor (AhR) and consequently regulate gene expression (Bostian and Eoff 2016).

An interesting study by Schroecksnadel et al. (2007) analyzed 146 patients suffering from a various kind of malignancies (mainly gastrointestinal tumors, hematological malignancy, gynecological neoplasms, and lung cancer). Fifty-four subjects were depressed and had to take antidepressant medication. Enhanced tryptophan degradation, measured by lower tryptophan levels and increase in kynurenine concentrations and K/T ratio, was related to a diminished quality of life (QoL), assessed by self-reported scores (from 1 to 5). This result emphasizes the role of immune-mediated tryptophan degradation in cancer-induced QoL deterioration, but, surprisingly, QoL was not significantly associated with depression. Nonetheless, the study did not directly measure depression status or antidepressant medication in relation to kynurenine pathway, leaving some questions open for future research.

Finally, plasma biomarkers of inflammation and kynurenine pathway activity are independent predictors of mortality due to cancer and the latter can be used as a prognostic factor (Zuo et al. 2016). In particular, even at the early stage of cancer, IDO activity is enhanced (Lyon et al. 2011) and such activity, in the vast majority of studies, has been associated with a poorer prognosis (Godin-Ethier et al. 2011; Gostner et al. 2015). Moreover, IDO activation may be linked to the development of cancer-related fatigue and thus to its debilitating consequences (Kim et al. 2015).

In their study on women with breast cancer, Lyon and colleagues (Lyon et al. 2011) found significant differences in tryptophan degradation, expressed in an enhanced IDO activity, between patients with early-stage breast cancer and healthy controls. One important consideration from the authors is that this could be relevant to the development of neuropsychiatric symptoms, including depression. As it is quite clear that tryptophan metabolism is critical in both depression and cancer, the assumption that in patients suffering from various types of cancer the development of depression might be associated to immune activation, especially to immune-mediated IDO activation, has gained more and more attention (Kurz et al. 2011). However, this hypothesis remains quite understudied. In Table 1, we have briefly summarized cancer types where alterations in kynurenine pathway have been demonstrated, together with prevalence rates of depression, assessed via diagnostic interviews or by self-reported questionnaires.

**Table 1:** Kynurenine pathway in cancer and rates of depression

Type of cancer	Kynurenine pathway alterations	Studies (*for kynurenine pathway alterations*)	Depression prevalence rates
Oropharingeal cancer	High IDO expression	Laimer et al. 2011	22–57%^1–2^
Pancreatic cancer	High IDO1 expression, high K/T ratio	Santhanam et al. 2016; Zhang et al. 2017; Huang et al. 2018	33–50%^1–2^
Breast cancer	High IDO and TDO expression	Lyon et al. 2011; Isla Larrain et al. 2014; Heng et al. 2016; Greene et al. 2018	1.5–46%^1–2-3^
Brain tumors	Increased IDO activity (high K/T ratio and QUIN/KYNA ratio)	Adams et al. 2014; Bostian and Eoff 2016	15–44%^4–5^
Lung cancer	High IDO expression, TDO2 activation	Hsu et al. 2016; Tang et al. 2017	3–44%^2–3^
Thyroid carcinoma	High IDO1 expression	Moretti et al. 2014	up to 36%^5^
Gynecological cancer	Increased IDO activity (high kynurenine and K/T ratio)	De Jong et al. 2011	12–26%^1–2-3^
Gastrointestinal cancer	High IDO1 expression	Santhanam et al. 2016	11–25%^1–2^
Hematological malignancies	High IDO expression	Hourigan and Levitsky 2011	1–25%^1–2-3^
Kidney cancer	High IDO1 expression, high K/T ratio	Van Gool et al. 2008; Lucarelli et al. 2017; Trott et al. 2016	6–24%^5–6^
Melanoma	High IDO and TDO expression	Capuron et al. 2003; Meng et al. 2017; Pilotte et al. 2012	4–20%^3–7^
Prostate cancer	High IDO expression	Feder-Mengus et al. 2008	15–19%^8^

^1^(McDaniel et al. 1995)

^2^(Massie 2004)

^3^(Krebber et al. 2014)

^4^(Richter et al. 2015)

^5^(Hartung et al. 2017)

^6^(Thekdi et al. 2015)

^7^(Kasparian 2013)

^8^(Watts et al. 2014)

## Methods

A systematic review was performed using PubMed, PsycINFO, Ovid MEDLINE ®, Embase, CINAHL, and ScienceDirect searches through December 2018. We searched for combinations of the following MeSH search terms: 1st category: “kynurenine” or “kynurenine pathway”; 2nd category: “cancer” or “neoplasm” or “tumor”; 3rd category: “depression” or “depressive disorder” or “mood disorder.”

**Fig. 1:**
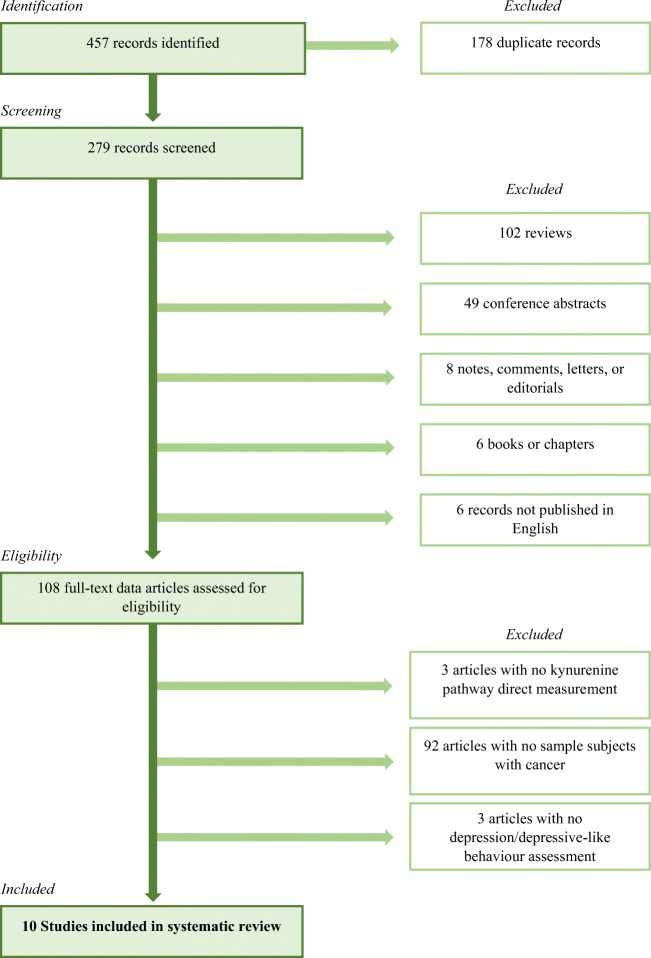
Systematic review flowchart

As explained in the flowchart (Fig. 1), the electronic search returned 457 records containing at least one term from each category. We identified 279 papers, excluding duplicates. We screened all titles and abstract; we excluded reviews, conference abstracts, notes, comments, letters, editorials, chapters, and books; and we included only English language published data papers. We then assessed 108 full-text data articles for eligibility. We retrieved the studies if they met the following inclusion criteria: (1) specifically measure kynurenine pathway alterations; (2) examine an animal or human sample of subjects with cancer; and (3) directly assess depression or depressive-like behavior. As a result, we finally reported ten studies specifically examining this relationship, listed in Table 2.

**Table 2:** List of studies specifically examining the role of the kynurenine pathway in linking depression and cancer

Author	Type of study	Type of cancer
Bannink et al. 2007	Clinical study	Melanoma; renal cell carcinoma
Barnes et al. 2018	Case report	Pancreatic adenocarcinoma
Bosnyák et al. 2015	Clinical study	Brain tumor
Botwinick et al. 2014	Clinical study	Pancreatic adenocarcinoma
Capuron et al. 2003	Clinical study	Malignant melanoma
Hüfner et al. 2015	Clinical study	Breast cancer
Lyon et al. 2018	Clinical study	Breast cancer
Norden et al. 2015	Animal study	Colon adenocarcinoma
Pertl et al. 2013	Clinical study	Breast cancer
Van Gool et al. 2008	Clinical study	Renal cell carcinoma

### Is kynurenine pathway the potential link between depression and cancer?

We have searched for both preclinical and clinical evidence of the role of the kynurenine pathway in linking depression and cancer. In particular, we investigated how the tryptophan shunt towards formation of kynurenine could be a key pathophysiological mechanism in the development of depressive symptoms during cancer.

An animal study on adult mice with a model of tumor growth (colon adenocarcinoma) was carried out by Norden and colleagues (Norden et al. 2015). They confirmed a neuro-inflammatory state associated with cancer and depression. In particular, tumor-bearing mice showed increased brain expression of pro-inflammatory cytokines, tumor-induced fatigue, and depressive-like behaviors. However, tryptophan metabolism was found not to be significantly altered, with no differences in IDO and only slight increases in KMO expression. Moreover, fluoxetine administration at a relatively low dose (3 mg/kg/day) in tumor-bearing mice reduced depressive-like symptoms, with no effect on tumor growth, muscle wasting, fatigue behavior, or cytokine and kynurenine pathway enzymes expression in the brain, suggesting that depressive symptoms may be treated as independent. It is still not excluded that administration of fluoxetine may act upstream in the pathway, by altering pro-inflammatory cytokine expression in the brain. In fact, previous studies have shown anti-inflammatory effects of fluoxetine by decreasing pro-inflammatory cytokines (Baumeister et al. 2016), with also a direct effect on the brain (Lim et al. 2009), supporting the need for further research.

As far as human studies are concerned, pancreatic cancer is known to have a particularly high prevalence rate of depression (Breitbart et al. 2014, Table 1), and so, it is one of the most studied. A case report was recently presented by Barnes et al. (2018) on a patient with pancreatic adenocarcinoma and a new-onset clinically assessed severe depression. The authors found markedly elevated levels of IDO1, IDO2, and TDO in patient’s resected tumor specimen. It has been hypothesized that kynurenine pathway is a biological basis for development of depression in this specific malignancy, together with alterations of other inflammatory pathways (Bettison et al. 2018). A study was conducted to specifically test this hypothesis (Botwinick et al. 2014), in which 17 patients with pancreatic adenocarcinoma were recruited and assessed for the presence of depressive symptoms, measured with the Beck Depression Inventory (BDI) scale. Findings indicated a negative correlation between mood scores and the plasma ratio between KYNA (already mentioned to have a neuroprotective effect) and tryptophan (KATR), while a positive correlation was found between tumor extension (tumor size and nodal involvement) and plasma kynurenine levels. The correlation between KATR and depression scores remained significant after controlling for the percentage of positive lymph nodes and maximal primary tumor diameter. Thus, the production of kynurenines (IDO metabolites) seems to be involved in the pathway between pancreatic cancer and depression, although a properly causal relationship is not demonstrated.

As we discussed, the role of immune activation in linking depression and cancer was hypothesized also in other types of cancer, such as breast cancer (Lyon et al. 2011). A clinical study assessed the levels of tryptophan metabolites in a sample of 154 subjects divided in 4 subgroups: suffering from breast cancer, depression, both, or neither (Hüfner et al. 2015). In breast cancer patients, depression was diagnosed assuming a ≥ 8 cutoff at the Hospital Anxiety and Depression Scale (HADS) or an ongoing treatment with antidepressant medications, while non-breast cancer patients were clinically diagnosed as depressed according to International Classification of Diseases (ICD-10) criteria. Significantly higher neopterin levels (considered as a marker of cellular immune system activation) were found in patients suffering from depression and anxiety, suggesting a pro-inflammatory state of type 1 T helper lymphocytes (Th1). Moreover, K/T ratio was significantly associated with breast cancer, state anxiety, and their interaction, with highest values in patients suffering from both conditions. Finally, the phenylalanine/tyrosine ratio, considered a measure of inflammation (Wannemacher et al. 1976), was higher in the comorbid cancer and depression subgroup. Interestingly, an excess of phenylalanine could interfere with serotonin production by competing with tryptophan for the same active transport channel to cross the blood-brain barrier (Linden et al. 2016). In a different study conducted in 61 breast cancer patients (Pertl et al. 2013), the authors did not find an association between kynurenine pathway alterations and depressive symptoms, assessed here too with HADS score, before chemotherapy. Still, such alteration of the pathway was found to be related to cancer treatment, as we will discuss below in the section “Implication for intervention.”

Finally, we have already mentioned neuroimaging alterations due to kynurenine pathway activation in depressed patients (Savitz et al. 2015a; Meier et al. 2016). Further analyses were performed more specifically in cancer-related depression. Bosnyák and colleagues utilized 11C-alpha-methyl-L-tryptophan (AMT)-positron emission tomography (PET) scanning on 21 patients with brain tumor without a previous history of clinical depression, assessing the presence of depressive symptoms through BDI scores. Seven patients showed clinical levels of depression (BDI score > 13) with associated abnormalities of tryptophan transport and metabolism. As the authors suggest, these modifications indicate a possible imbalance between the serotonin and kynurenine pathways. Moreover, this could serve as a molecular imaging marker of brain tumor–associated depression (Bosnyák et al. 2015).

### Potential confounders in this association

Despite this evidence, the symptoms overlap between cancer and depression could bias the assessment of depressive symptoms in oncologic patients, as we mentioned above, leading to misdiagnose depression. On the other hand, many biological modifications strictly related to cancer, such as the increased energy demand, or the biological impact of cancer treatments, could be a trigger for the development of neurovegetative symptoms, regardless of kynurenine pathway activation and the presence of clinical depression.

We already discussed that symptoms like fatigue in oncologic patients could be associated with kynurenine pathway activation (Kim et al. 2015; Norden et al. 2015). However, their precise etiological and pathophysiological mechanisms are still unknown. Even though it is conceivable a crucial role of inflammation, they could also involve other factors in a complex interaction between immune, endocrine, and nervous systems (Berger et al. 2015; Saligan et al. 2015). Interestingly, energy demand and fatigue, although obviously related, should be considered as distinct symptoms (Eshragh et al. 2017), suggesting that several different factors contribute to the development of neurovegetative symptoms during cancer. In addition, it should be considered that cancer and cancer therapy could very often be related to significant levels of stress. In this regard, it has been demonstrated that stress is, in turn, associated with kynurenine pathway activation (O’Farrell and Harkin 2017) through interactions between the autonomic nervous system and the immune system (Won and Kim 2016). Even stress could therefore contribute to the relationship investigated in this paper. Thus, there is a vast variety of symptoms and clinical conditions, all connected to each other, that could be related directly or indirectly to the kynurenine pathway activation and hence to its consequences. Accordingly, we believe that an accurate diagnosis of clinical depression with validated and replicable tools is crucial to disentangle the different contribution of cancer and depression to the overall clinical picture and to better understand the underlying mechanism of such comorbidity. We will debate below the additional confounding effects of chemotherapy.

The results we discussed confirm an alteration in the kynurenine pathway in both cancer and depression, with also evidence of a mutual interaction. However, a potential role of such alteration in causing depressive symptoms during cancer is still uncertain, due to discrepancies between the results, and the paucity of studies.

### Implication for intervention

### IFN-alpha treatment

Although it represents today only a marginal treatment for hepatitis C, the IFN-alpha therapy is still used for the treatment of renal cancer and melanoma, and it is the perfect evidence of a cancer therapy that challenges the immune system just like cancer itself. As a consequence, IFN-alpha therapy can lead to depression. Indeed, IFN-alpha therapy is widely known to be associated with high rates of psychiatric side effects, particularly depressive symptoms (Felger et al. 2016; Hepgul et al. 2016).

Again, this relationship could be due to an over-stimulation of kynurenine pathway, especially via IDO activation, caused by inflammatory cytokines (Russell et al. 2018). A preclinical study by Fischer et al. (2015) found that IFN-alpha administration induced depressive symptoms (measured with the forced swim test) in rats and an increased K/T ratio. However, while imipramine administration seemed to improve depressive-like behavior without acting on the tryptophan-kynurenine pathway, treatment with celecoxib seemed to reverse both depressive symptoms and the K/T ratio. Similarly, in a study on patients with malignant melanoma treated with IFN-alpha, Capuron and colleagues (Capuron et al. 2003) demonstrated that patients developing depression, diagnosed according to DSM-IV criteria, showed greater increases in kynurenine, neopterin, and K/T ratio. In these patients, the SSRI antidepressant paroxetine could attenuate the behavioral consequences of IFN-alpha by acting more on the tryptophan availability than on kynurenine or neopterin response. These findings suggest that, despite the psychiatric side effects commonly related to IFN-alpha treatment could be mediated by an activation of the kynurenine pathway, therapy with antidepressant may not be effective on this pathway, but rather improve depressive symptoms in other ways.

However, there are also studies disconfirming the link between the kynurenine pathway activation induced by IFN-alpha and the onset of depression. It has been reported that cancer patients treated with IFN-alpha exhibited an activation of the kynurenine pathway with consequent depletion of tryptophan levels. IFN-alpha treatment was found to alter tryptophan metabolism increasing its peripheral degradation, but this action seemed to be independent from psychiatric side effects. A study on patients with either melanoma or renal cell carcinoma found that IFN-alpha treatment altered tryptophan levels without inducing psychiatric side effects, assessed through clinical interviews and observer-based rating scales (MADRS), in addition to a self-reported questionnaire, the Symptom Check List-90 (SLC-90) (Bannink et al. 2007). In a further study on patients with renal cell carcinoma, IFN-alpha action on kynurenine pathway was proved to cause an imbalance between neurotoxic and neuroprotective metabolites of kynurenine, as seen in an increase in neurotoxic compounds, even though not always related to psychopathological symptoms, measured by the Mini-International Neuropsychiatric Interview (MINI) and the SCL-90 (Van Gool et al. 2008). These findings may be due to the small sample sizes in both studies and to differences in cancer types, and highlight the need for more studies investigating the psychiatric implication of treatment with IFN-alpha in cancer.

### Chemotherapy effect on kynurenine pathway

Inflammatory dysfunction is not only related to cancer itself, but also to its treatment. The improvements in cancer surviving we have assisted to in the last decades (Jemal et al. 2017) have not always been followed by an improvement in psychiatric comorbidities, like depression, which remain highly prevalent among cancer patients. It has been hypothesized, and confirmed by a number of studies, that behavioral symptoms in cancer patients could be related to neurotoxic effects of cancer treatment, especially chemotherapy, in a synergic action between cancer and its treatment (Wardill et al. 2016; Santos and Pyter 2018). Indeed, although inflammation, as we discussed, is pivotal in cancer development (Munn 2017), several of the most common and widely used chemotherapeutic agents, like cisplatin, paclitaxel, 5-fluorouracil, and doxorubicin, have a demonstrated pro-inflammatory effect (Vyas et al. 2014). A strong tumor-associated immune response could be initiated by cancer therapy, and an inhibition of therapy-induced inflammation may hence improve cancer outcome (Grivennikov et al. 2010). However, an inflammatory response has not been observed with every chemotherapeutic compound and, to date, studies on the relationship between chemotherapy and the kynurenine pathway are insufficient and inconsistent.

Nonetheless, this potential pro-inflammatory effect of chemotherapeutic agents may lead to development, worsening, and persistence of depressive symptoms, through the activation of the kynurenine pathway. This evidence was supported by Pertl et al. (2013). In their study, the authors did not find any correlation between depression, assessed using the HADS, and kynurenine pathway activity before chemotherapy. Still, they found an enhanced inflammatory activity after cancer treatment. Moreover, intra-individual changes in pre- and post-treatment kynurenine levels predicted changes in depression over time, suggesting that increased kynurenine pathway activation may contribute to depressive symptoms aggravation in patients treated for cancer. Furthermore, alterations of kynurenine pathway were found after chemotherapy in a recent pilot study on 19 women with early-stage breast cancer (Lyon et al. 2018). The authors analyzed global metabolites post-chemotherapy and observed significantly higher kynurenine levels and K/T ratios, together with increased levels of depressive symptoms, assessed again through HADS score. Nonetheless, neither kynurenine nor tryptophan levels were associated with depression. These results suggest that chemotherapy may increase the kynurenine pathway activation and contribute to depressive symptoms in patients treated for cancer, pointing out the indirect effect of chemotherapy on maintaining depressive symptoms, even though the precise mechanism is still unknown and needs further investigation.

### IDO inhibitors

Given IDO crucial role in tumorigenesis and tumor progression, molecules with the ability to inhibit IDO activity have increasingly gained attention in immuno-oncology. In particular, a number of studies are now investigating whether inhibitors of IDO1 may improve patient responses to anti-PD1 immune checkpoint therapy in patients with advanced melanoma (Muller et al. 2018). Immune checkpoints are molecules that regulate the immune system by modulating immune tolerance and preventing uncontrolled immune responses. Specific checkpoint inhibitors are therefore emerging as new pharmacological targets to enhance cancer treatment. In particular, PD1 (programmed cell death protein 1) is a cell surface protein that is involved in immune suppression, and its ligand (PD-L1) is highly expressed in several cancers (Francisco et al. 2010; Wang et al. 2016b). Thus, some cancers use this route to block the immune response and continue growing. For this reason, PD1 and PD-L1 inhibitors, known as checkpoint inhibitors, are emerging as a novel immunotherapy for cancer (Alsaab et al. 2017). An important limit for this new treatment strategy, however, is that cancer can still maintain an immunosuppressive microenvironment by activating IDO and the kynurenine pathway (Toulmonde et al. 2018). Consequently, IDO inhibition may be advantageous to improve response rates to anticancer immunotherapies, such as checkpoint inhibitors.

A large number of molecules to date have been tested as IDO inhibitors and utilized in clinical trials as anticancer compounds, mainly in combinatorial regimens and in association with other chemotherapeutic agents, with more promising results than their administration as a single drug. However, none of these molecules is up to now approved for clinical use (Zhang et al. 2018). So far, the most studied molecules are IDO1 inhibitors, but there has been a recent and rising interest in IDO2 and TDO inhibitors as well. The 1-methyltryptophan, a competitive inhibitor of IDO1 (and IDO2) that exists as a mixture of chiral isoforms (i.e., 1-methyl-D-tryptophan (1-MDT) also known as indoximod and NLG8189), as well as second-generation IDO1 inhibitors (such as the orally available agent epacadostat (INCB024360) and NLG919), and IDO1-targeting vaccines, were studied clinically and preclinically in cancer patients. So far, other IDO1 inhibitors, including 1-methyl-L-tryptophan, methylthiohydantoin tryptophan, brassinin and derivatives, annulin B and derivatives, and exiguamine A and derivatives, as well as INCB023843, are in development (Vacchelli et al. 2014); other drugs include benzofuranquinones and annulin A analogues (Carvalho et al. 2014), just to name a few. 1-MDT is probably the most well studied and it is involved in some encouraging clinical trials (Godin-Ethier et al. 2011). Up to December 2018, 22 clinical trials involving IDO inhibitors and one involving an IDO targeting vaccine were ongoing, according to “ClinicalTrials.gov” results. Most of them were in phase I or II, four were in phase III. Only three studies, all in phase I, were evaluating the safety, tolerability, and pharmacokinetics of a single IDO inhibitor; the others were evaluating the efficacy of a combination of these compounds with chemotherapy or immunotherapy, combining them for example with immune checkpoint inhibitors.

A recent paper raised the question about the real effectiveness of IDO inhibitors, starting from the evidence of the surprising failure of ECHO-301, a large phase III clinical trial (Garber 2018). The trial involved roughly 350 patients receiving epacadostat (INCB024360), an IDO1-selective enzyme inhibitor we mentioned above, with pembrolizumab, an anti-PD1 humanized monoclonal antibody, for melanoma treatment. The combination therapy missed its first primary endpoint of improving overall survival, compared to pembrolizumab monotherapy. Nevertheless, this discouraging result should be analyzed with caution, avoiding definitive judgements. The negative outcome could reflect uncertainties about particular aspects of the trial and raises some questions. For example, whether the target was adequately inhibited, or if there was a mechanistic rationale for the combination tested, or again, according to preclinical data, whether a broader-spectrum approach, inhibiting both IDO and TDO, would have been better (Muller et al. 2018). Moreover, other IDO inhibitor molecules, with potential different chemical properties, could reveal their efficacy in cancer treatment and other combination therapies will possibly be refined. The use of this new therapeutic target may also improve the management of depression during cancer. However, at this stage of research, the efficacy of IDO inhibitors in treating both cancer and depression can only be hypothesized. Indeed, the management of cancer-related depression still relies on antidepressant, which may not only enhance monoamine availability, but also act on inflammatory pathways.

## Conclusion

Given the evidence to date, it is conceivable that the kynurenine pathway may be one of the biological links between depression and cancer. However, more studies are required to further analyze the role of kynurenine pathway in the development of depression in different types of cancer, which could be related to diverse biological mechanisms. Similarly, the effect of specific cancer therapies on the development and persistence of depressive symptoms should be examined in future research. From a clinical point of view, targeting IDO activity appears to be a promising treatment approach for cancer comorbid with depression. Therefore, not only the anti-tumoral but also the antidepressant efficacy of IDO inhibitors should be assessed during future clinical trials.

## Electronic supplementary material

ESM 1 (JPG 2298 kb)

## Abbreviations

1-MDT: 1-methyltryptophan
AhR: Aryl hydrocarbon receptor
AMT: 11C-alpha-methyl-L-tryptophan
BDI: Beck Depression Inventory scale
CNS: Central nervous system
CRP: C-reactive protein
DSM: Diagnostic and Statistical Manual of Mental Disorders
HADS: Hospital Anxiety and Depression Scale
HAO: 3-Hydroxyanthrenilic acid dioxygenase
ICD: International Classification of Diseases
IDO: Indoleamine 2,3 dioxygenase
IFN: Interferon
IL-6: Interleukin 6
K/T ratio: Kynurenine-to-tryptophan ratio
KATR: Kynurenic acid-to-tryptophan ratio
KYNA: Kynurenic acid
MADRS: Montgomery–Åsberg Depression Rating Scale
MINI: Mini-International Neuropsychiatric Interview
MDD: Major depressive disorder
mPFC: Medial prefrontal cortex
MRI: Magnetic resonance imaging
NAD+: Nicotinamide adenine dinucleotide
NK cells: Natural killer cells
NMDA: N-methyl-D-aspartate
PD1: Programmed cell death protein 1
PD-L1: Programmed death-ligand 1
PET: Positron emission tomography
QoL: Quality of life
QUIN: Quinolonic acid
QUIN/KYNA: Quinolonic acid-to-kynurenic acid ratio
SCL-90: Symptom check list-90
SSRI: Selective serotonin reuptake inhibitor
TDO: Tryptophan 2,3 dioxygenase
Teff: Effector cytotoxic CD8+ T cells
Th1: Type 1 T helper lymphocytes
TRD: Treatment-resistant depression
Tregs: Regulatory T cells
