# Supplementary figures and images for: Inflammation in cancer and depression: a starring role for the kynurenine pathway
Source: Psychopharmacology (Berl). 2019 Feb 26;236(10):2997–3011. doi: 10.1007/s00213-019-05200-8 (PMC6820591; doi:10.1007/s00213-019-05200-8)

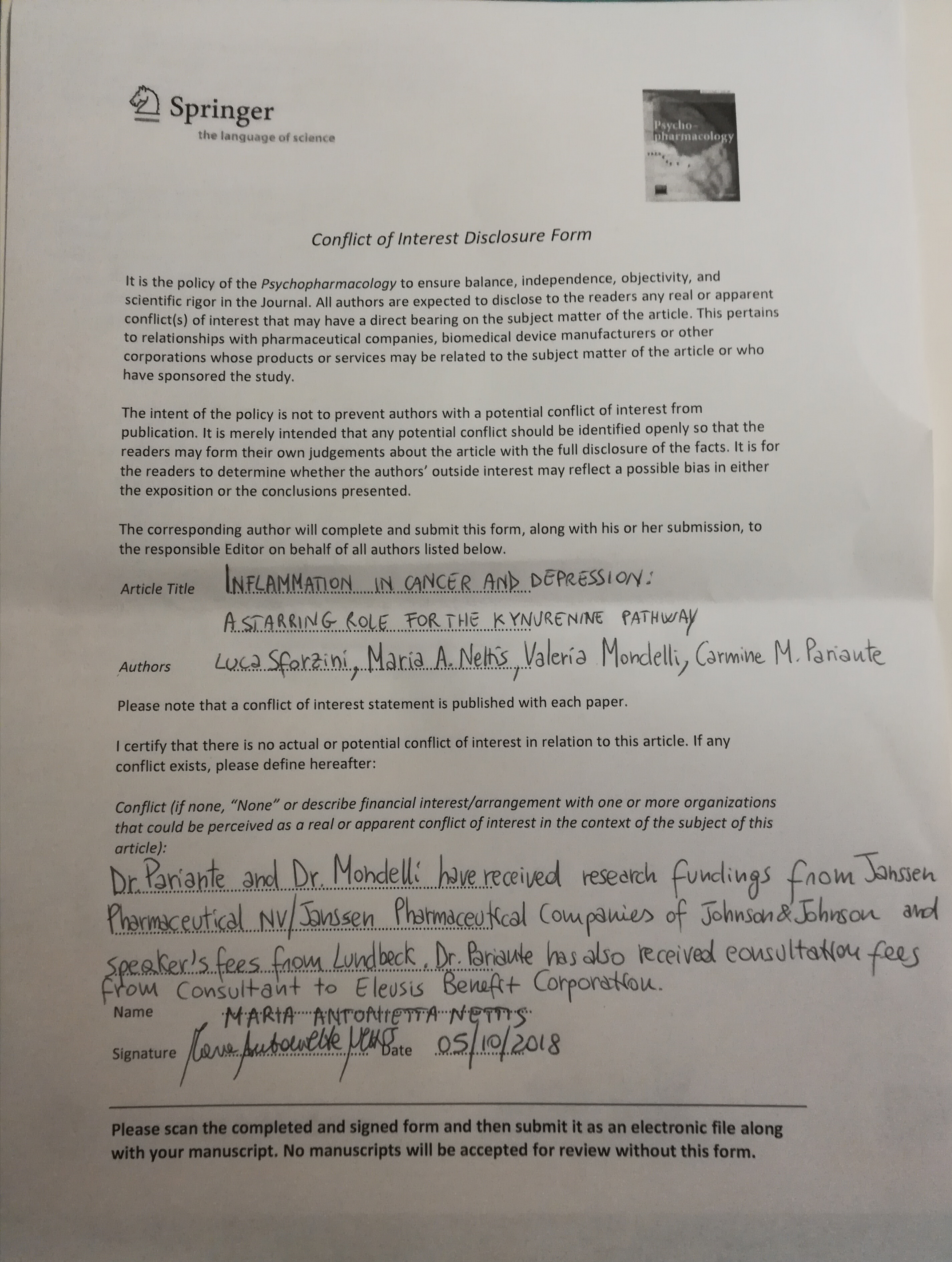

Supplement: Supplementary file 1 — (JPG 2298 kb) [file 213_2019_5200_MOESM1_ESM.jpg]
